# Antibacterial activity of pigment extracted from bacteria isolated from soil samples

**DOI:** 10.1186/s13104-024-06834-4

**Published:** 2024-06-19

**Authors:** Shashi Bhushan Chaturwedi, Swostika Mainali, Richa Chaudhary

**Affiliations:** Department of Microbiology, D.A.V. College, Lalitpur, Nepal

**Keywords:** Antimicrobial susceptibility assay, *S. Aureus*, Carotenoids, Pyocyanin

## Abstract

The purpose of this study was to evaluate antibacterial activity of pigment extracted from bacteria, isolated from soil samples. During the study, 20 soil samples were collected from different areas (forest, agriculture fields, river sides and dumping sites) of Kathmandu and Lalitpur districts which were processed for isolation of pigment producing bacteria by spread plate technique. The pigmented bacterial isolates were identified and enriched in nutrient broth. Then, pigment was extracted in 95% methanol as solvent, which was further characterized using UV-Vis Spectrophotometric and TLC analysis. The obtained crude pigment extract was processed to carry out the antimicrobial susceptibility assay using agar well diffusion method. Out of 13 total pigmented bacteria isolates, four different colored pigmented bacterial isolates (S4O, S11Y, S14P and S17G) which produced efficient pigment on nutrient agar were chosen and they were further processed. Among these isolates, S4O was identified as *Staphylococcus aureus*, S11Y was identified as *Micrococcus luteus*, S14P was identified as *Micrococcus roseus* and S17G was identified as *Pseudomonas aeruginosa* respectively. On characterization using UV-Vis Spectrophotometric and TLC analysis, the pigment extracted from isolates S4O, S11Y and S14P were found to be Carotenoids and from isolate S17G was found to be Pyocyanin in nature. The maximum antibacterial activity was shown against *Staphylococcus aureus* from all the four pigments extracts. The green color pigment extract from isolate S17G was found to be most effective against all the Gram-positive and Gram-negative test bacteria. This study suggests that these pigment extracts from pigmented bacteria may have beneficial antibacterial roles that can be exploited in controlling unwanted bacterial growth.

## Introduction

A pigment is a material that changes the color of reflected or transmitted light as the result of wavelength-selective absorption. This physical process differs from fluorescence, phosphorescence, and other forms of luminescence, in which a material emits light [1]. Natural pigments are sourced from ores, insects, plants, animals and as well as from microbes. Bacteria that produce pigment are called as Chromogenic bacteria [2]. The pigments production in bacteria is important for protection against ultraviolet radiation, oxidantion stress, extreme temperature and desiccation. Pigments are also known to act as bacterial shields against some natural antimicrobial compounds produced by other microorganisms [3]. Some bacteria produce pigment as part of their normal metabolism with color of different shades. Bacteria are known to produce various pigments like Carotenoids and its derivatives (β-carotein, Astaxanthin, Canthaxanthin, Zeaxanthin), Prodigiosin, Pyocyanin, Melanin, Violacein etc [4, 5]. Microbial pigments are the characteristic feature of some bacteria which may be useful in identification as pigmented bacteria will form cultures that exhibited some color Production of pigments can be extracellular or intracellular depending on the micro-organisms. Some pigments are water soluble they leak out of cells and diffuse through the water-based culture medium which causes the medium to change color. Other pigments are water insoluble they remain within the cells, so only the colony becomes colored. It has been proved that only aerobic and facultative anaerobic bacteria are pigmented because molecular oxygen is essential for pigmentation [6].

The use of microorganisms for pigment production has several advantages including easy propagation and wide strain selection, availability of the cultivation technology, easy downstream processing and more cost effective [7]. The extraction and characterization of microbial pigments from pigmented microbes are easier than non-pigmented microbes, whose identification and compound characterization is hard and time-consuming. In addition, the collection of microorganisms is sustainable and has no negative impact on the environment [8].

Bacterial pigment production is now one of the emerging fields of research to demonstrate its potential for various applications. These pigments are not only used for coloring agents in the cosmetic and food industry but have also been reported to possess biological properties such as antimicrobial, antiviral, antioxidant and anticancer activities. Out of all the secondary metabolites having antimicrobial activity, pigments are the least studied group [9].

The exploitation of new antimicrobial agents from natural resources is considered as an important task particularly in developing countries where the threat of drug resistance in microorganism is more. Thus, the bacteria pigment as secondary metabolites are seen to be a promising agent in this regard. This study aimed at isolation and identification of bacteria which are having pigment producing ability from natural habitat like soil. The pigment from the isolates is processed for production, extraction, and evaluation of the antimicrobial activity.

## Methods

### Sampling sites

Total 20 soil samples were collected from different areas (forest, agriculture fields, river sides and dumping sites) of Kathmandu and Lalitpur districts which were processed for isolation of pigment producing bacteria.

### Sample processing

Bacteria present in the samples were isolated by spread plate technique on NA medium. The isolated pigment producing bacteria were streaked on sterile NA plate to obtain pure culture. The pure pigmented bacterial cultures were grown on nutrient agar slant and maintained at 2–4 °C temperature in refrigerator. Out of total isolates, different pigment producing bacteria which produced efficient pigment on NA agar were chosen and studied further.

### Identification of pigmented isolates

Bacterial isolates were identified as described in the Bergey’s manual of systemic bacteriology which involves colony characterization, cell morphology and biochemical tests.

### Enrichment for pigment production

A pre-culture was developed in 5 ml of NB medium by inoculating the broth with one loop full of selected bacteria and incubated at room temperature for overnight. The pre-culture was then used to inoculate (1 ml) in NB production medium. The approximate cell concentration was observed using the McFarland standard graph. Production of pigment was carried out in 50 ml nutrient broth in 100 ml conical flask and incubated at 37 °C for 48 h [10].

### Extraction of pigment

For extraction of pigment, the bacterial cells were first grown for 48 h in nutrient broth followed by centrifugation at 3000 rpm for 20 min. The pigment containing layer (pellet/ supernatant) was taken, mixed with 10 ml 95% (v/v) methanol for extraction [11]. The colored methanolic supernatant extract was collected and filtered through Whatman No. 1 filter paper. The filtrate was poured in an evaporating dish and incubated at 50 °C in hot air oven for 30 min to obtain a dry pigment extract, free of solvent. The mass was stored at 4 °C until further use.

### Spectrophotometric analysis

The pigment extract was then analyzed by scanning the absorbance in the wavelength region of 200–700 nm using UV-Vis spectrophotometer which helps to find out the maximum absorption spectra (ʎmax) by using 95% methanol as a blank [12].

### TLC analysis

About, 50 µl of methanolic pigment extracts were spotted on the baseline of the TLC plate with the help of pipettes and then allowed to dry. The TLC plates were then placed in a pre-saturated TLC chamber containing the mobile phase (methanol: chloroform in the ratio of 6:4). The Retention factor (Rf) values were determined using the formula mentioned below;

$$\text{Rf} = \frac{\text{Distance travelled by solute front (d)}}{\text{Distance travelled by solvent front (D)}}$$

### Antimicrobial susceptibility assay

The agar well diffusion method was performed to analyze the antibacterial activity against the test organisms. All the test microorganisms were obtained from the Microbiology Laboratory of D.A.V. College, Lalitpur. The pigment extract was prepared by dissolving 1000 mg of pigment extract into 1 ml of 10% methanol. Each well was filled with 50 µl of methanolic extract of bacterial pigments and 10% methanol was used as a negative control. After proper incubation at 37 °C for 24 h., zone of inhibition (ZOI) surrounding each well was measured in millimeters and noted.

### Data analysis

All the data were entered in Microsoft Excel and thus interpretation of the entered data was characterized in the form of diagram, graphs and tables for visual appreciation of different analytical aspects.

## Results

### Isolation of pigment producing bacteria

**Fig. 1:**
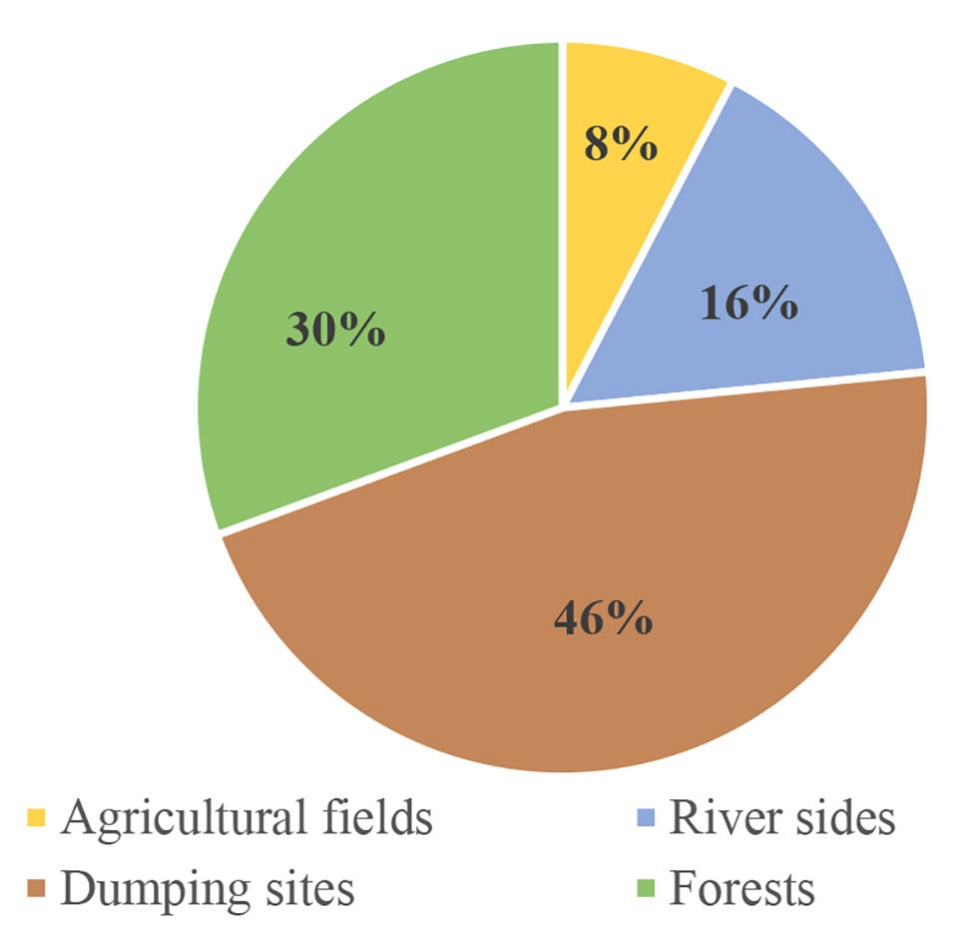
Distribution of pigment producing bacteria in different sampling sites

From 20 soil samples processed, pigmented bacteria were isolated in 9 (45%) soil samples with total of 13 isolates. Out of theses pigment producing bacterial isolates, higher percentage i.e., 46% (6) were isolated from dumping sites where as the least percentage of pigment producing bacterial isolates were 8% (1) from agricultural fields (Fig. 1).

**Fig. 2:**
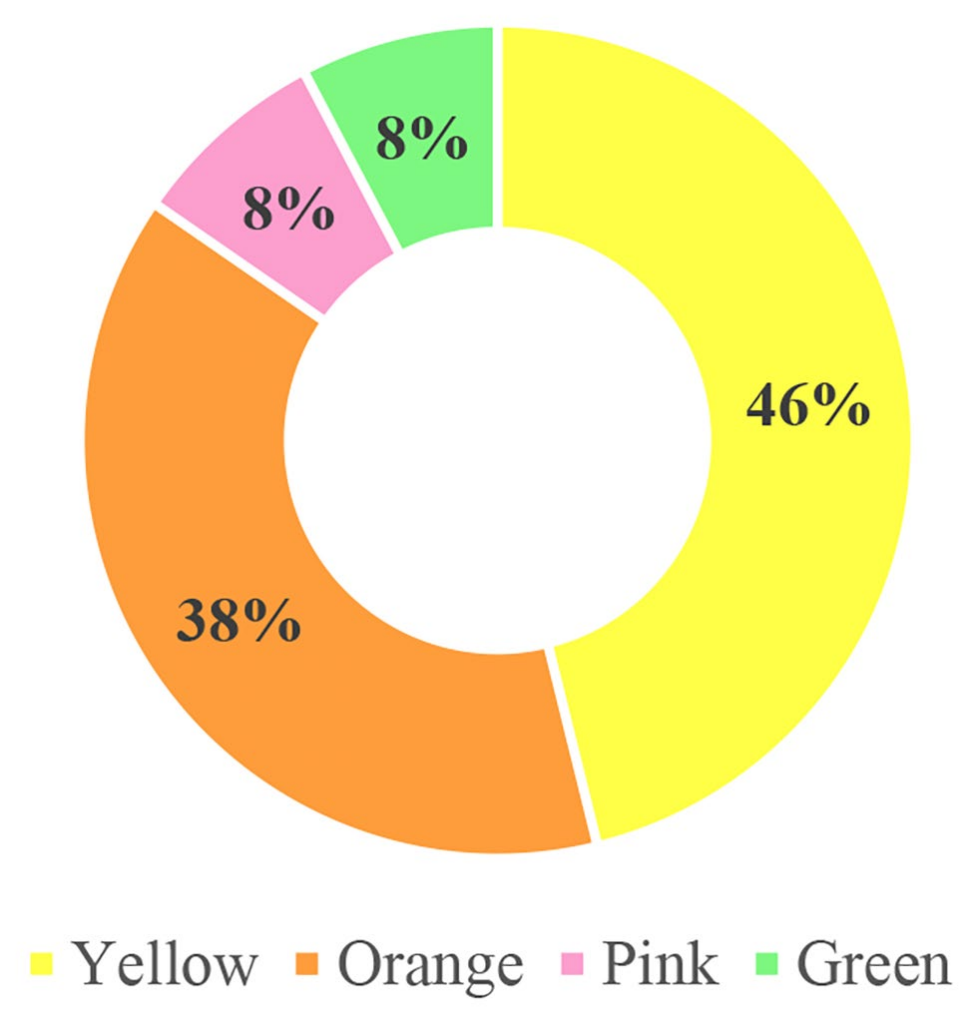
Percentage of different pigmented bacteria isolates from soil samples

Isolated bacteria showed different pigmentation. Out of 13 pigmented bacterial isolates, yellow pigment producers were highest i.e., 46% (6), followed by 38% (5) orange pigment producers. The least percentage was of pink and green pigment producer i.e., 8% (1) each among the isolates (Fig. 2).

### Identification of pigmented bacterial isolates

In this study, a total of four different color pigmented bacteria isolates which produced efficient pigment on nutrient agar were chosen and they were further processed. Among these, isolate S4O was identified as *Staphylococcus aureus*, isolate S11Y was identified as *Micrococcus luteus*, isolate S14P was identified as *Micrococcus roseus* and isolate S17G was identified as *Pseudomonas aeruginosa* respectively.

### Thin layer chromatography analysis

It is observed that tested pigments from isolates S4O, S11Y and S14P showed spot on TLC plates with Rf values 0.78, 0.88 and 0.65 respectively. These Rf value lies within standard carotenoids range i.e., 0.9 to 0.34 [13], therefore indicating the presence of carotenoids pigment. Likewise, the observed corresponding TLC Rf value was found to be 0.74 showing single blue color spot on TLC plates indicating the presence of pyocyanin pigment from isolate S17G.

### Spectrophotometric analysis

**Fig. 3:**
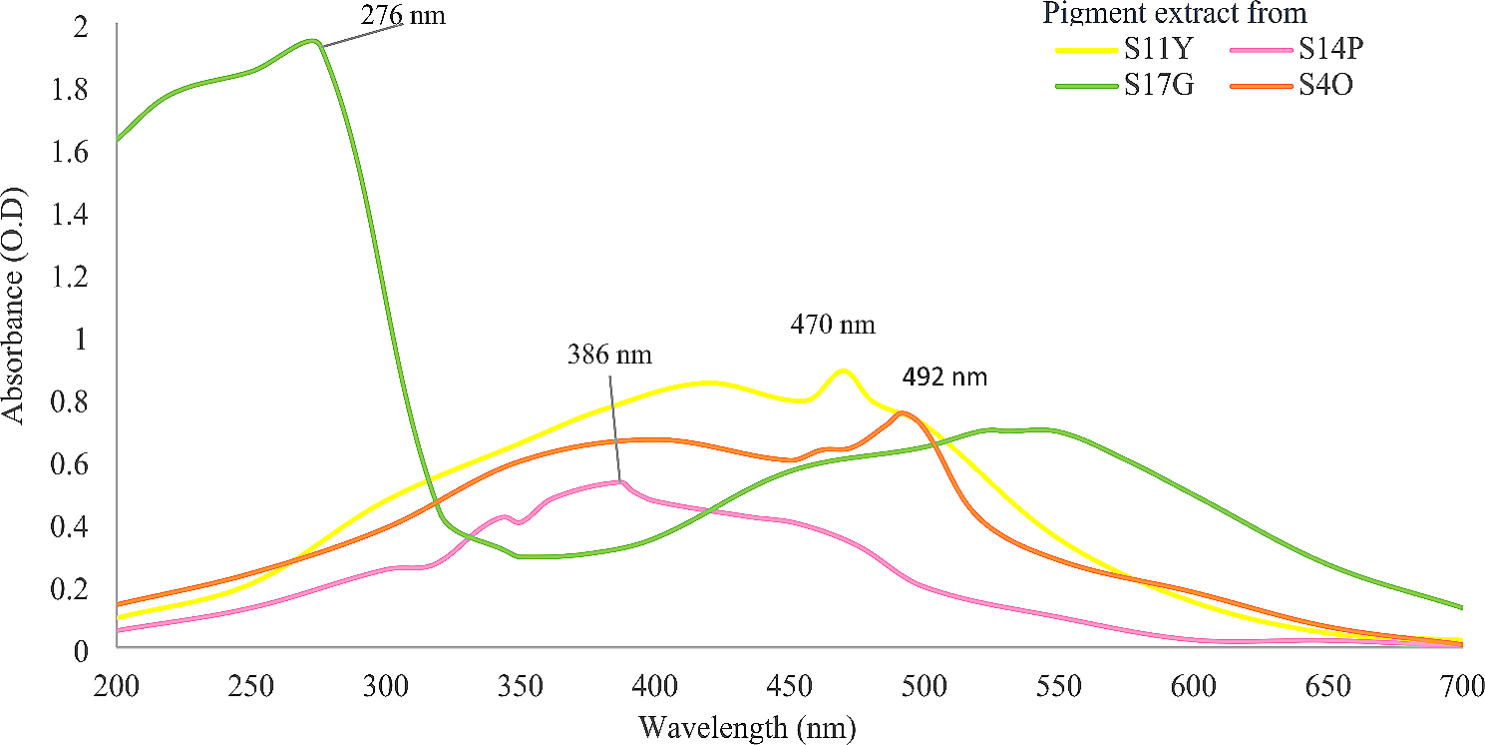
UV -Vis spectrophotometric analysis of pigment extract showing maximum absorbance (λmax)

As shown in Fig. 3, the green pigment from isolate S17G was found to be pyocyanin showing maximum absorbance at 276 nm. The extracted pigments from isolates S4O, S11Y and S14P were found to be carotenoids as they showed maximum absorbance in the range of 492 nm, 470 nm and 386 nm respectively. Most of the carotenoids show peak between 400 and 500 nm ranges [14].

### Antibacterial activity of extracted pigments

Among the four pigment extracts, the green color pigment from isolate S17G was found to be most effective against all the Gram-positive and Gram-negative test bacteria showing highest ZOI of 34 mm against *S. aureus*. It had maximum ZOI of 24 mm against *Bacillus* spp., 20 mm against *E. coli* and *K. pneumonia* each, 26 mm against *Proteus* spp. and 16 mm against *S.* Typhi respectively.

The maximum antibacterial activity was shown against *S. aureus* with ZOI of 20 mm from orange pigment extract from isolate S4O, ZOI of 16 mm from yellow pigment extract from isolate S11Y, ZOI of 18 mm from pink pigment extract from isolate S14Pand ZOI of 34 mm from green pigment extract from isolate S17G, respectively (Table 1). These pigments also showed significant antibacterial activities against the other bacterial test pathogens as well.

**Table 1:** Antimicrobial activity of 10% methanol extract of pigments against selected bacterial strains

Isolate	Pigment color	Zone of inhibition (mm) against test microorganisms					
		S. aureus	Bacillusspp.	E. coli	K. pneumoniae	Proteusspp.	S.Typhi
S4O	Orange	20	ND	ND	ND	ND	ND
S11Y	Yellow	16	ND	6	ND	8	4
S14P	Pink	18	ND	10	ND	8	8
S17G	Green	34	24	20	20	26	16

Note: ND- Not detected

## Discussion

Pigmented bacteria were isolated in 9 (45%) soil samples with total of 13 isolates. Out of theses pigment producing bacteria isolates higher percentage i.e., 46% (6) were isolated from dumping sites while least percentage of isolate was 8% (1) from agricultural fields. However, in terms of color of pigment, the yellow pigment producing colonies were found predominant i.e., 46% (6) of total isolates. Hereby, these results underscore that variability of pigmented bacteria is associated with the variable habitat of origin in the same geographical area.

For the extraction of crude microbial pigment from freshly grown bacterial culture various methods were used like centrifugation, vortexing, filtration and addition of 95% methanol so that cell gets lysed and pigment can be extracted. The methanol as solvent extracted most of the pigments from the cell and in addition coagulated the cellular debris to a sufficient degree that it enabled the recovery of pigment in clear filtrate.

The maximum absorbance of orange pigments from isolate S4O is at 492 nm, yellow pigment from S11Y is at 470 nm, and pink pigment from S14P is at 386 nm respectively. So on, the study of [15] characterized the yellow pigment synthesized by the *Micrococcus luteus* strain NCTC 2665 as carotenoid which showed maximum absorbance at 467 nm. A similar result was observed for carotenoid derivative canthaxanthin as the main pink pigment produced by *Micrococcus roseus* having λmax measured by spectrophotometer at 372 and 426 nm [16]. Based on the spectral analysis (λ-max) as the extracted pigment lies within the standard wavelength peak of carotenoids pigments i.e. between 300 and 500 nm [14] and comparison with similar findings, the pigments from isolates S4O, S11Y and S14P were identified as carotenoids. So on, the result of our study from isolate S17G with green pigment having maximum absorbance at 276 nm correlated with the work of [17] extracted blue-green pigment from *Pseudomonas aeruginosa* which showed a maximum absorbance at 252 nm. This peak indicates the presence of pyocyanin compound.

Furthermore, the extracted blue-green pigment from *Pseudomonas aeruginosa* subjected to Thin Layer chromatography using silica gel as a stationary phase and chloroform as a mobile phase with corresponding TLC Rf value 0.71 indicates the presence of pyocyanin pigment [17], which was similar to the green pigment from isolate S17G having Rf value 0.74. TLC also showed the presence of a pigment which migrated as colored component in the sheet with Rf 0.78 from orange pigment extract of isolate S4O, yellow pigment extract of isolate S11Y with Rf 0.88 and pink pigment extract of isolate S14P with Rf 0.64 respectively. Similar findings were reported by many researchers, such as standard carotenoid pigment production from *Micrococcus luteus* is reported to have Rf value of 0.85 [12]. The Rf value from orange pigment of *staphylococcus aureus* was found to be 0.38, and 0.69, thus characterized as carotenoid pigment staphyloxanthin [18]. As, the results of extracted pigments lies within the standard range of Rf values of carotenoids i.e., 0.92 to 0.34 [13], which indicates that these pigments are from carotenoid family.

In this study, the antimicrobial activity of extracted carotenoid pigment was found to be most effective against Gram-positive than Gram-negative bacteria. The reason behind this might be due to the difference in the cell structure as the Gram-negative bacteria possess outer membrane which can act as a barrier for antimicrobials to enter to the cells. However, the mode of action behind the antibacterial activity from these extracted bacterial carotenoids pigments is still unknown. The green color pigment from isolate S17G was found to be most effective against all the Gram- positive and Gram-negative test bacteria. Pyocyanin exhibits intracellular oxidant stress and initiates a redox cycle that results in production of reactive oxygen species such as hydrogen peroxide and superoxide, these compounds inhibit the growth of pyocyanin sensitive microbes [19]. As well, the difference in lipid content of cell wall of Gram-negative and Gram-positive bacteria might be associated with different level of sensitivity of test bacteria to pyocyanin [20]. The variation in antimicrobial activities might have been due to the differences in nature of pigment produced by isolated pigmented bacteria.

## Conclusion

This study suggests that bacterial isolates from local soil samples could synthesize pigments. The pigment extracted from isolated pigmented bacteria showed comparable antibacterial activity against both the Gram-positive as well as Gram-negative bacteria indicating antibacterial nature of the pigment. Thus, the pigment extracts can be used to control the growth of unwanted pathogens.

## Data Availability

All the required data and material of research is given in the manuscript.
